# Slipping and holding minds: A psychosocial analysis of maternal subjectivity in relation to childhood disability

**DOI:** 10.4102/ajod.v5i1.266

**Published:** 2016-07-26

**Authors:** Lisa Saville Young, Jessie Berry

**Affiliations:** 1 Department of Psychology, Rhodes University, Grahamstown, South Africa

## Abstract

**Background:**

This paper elucidates a methodological approach to interview text that tries to acknowledge the psychosocial nature of disability and thereby ensuring that empirical work in disability studies complements theoretical arguments already developed.

**Objectives:**

The aim of this study is to outline a psychosocial conceptualisation of maternal subjectivity in relation to childhood disability and to apply this conceptualisation as an analytic tool to segments of an interview with a mother of a child with physical and developmental disabilities.

**Method:**

Drawing on psychoanalysis and attachment literature alongside critical social psychology we take readers through the analysis of an interview extract with a particular mother. Through a fine grained analysis, we demonstrate the value of attending to the affective processes in and around the text rooted in the particular intersubjective exchange (‘here and how’) of the interview and the particular socio-historical context (‘there and then’) in which the mother, child and researcher are located.

**Findings:**

The reading draws attention to discourses that position this particular mother and her children in particular ways while also pointing to investments in these discourses such that these discourses are not purely social but play affective functions.

**Conclusion:**

This paper demonstrates the value of using multiple lenses to read the text, seeking to understand what is going on from within each lens (discursive/social, interpersonal, intrapsychic), while also seeking to disrupt this understanding as we take up the position of a different lens. This approach enables us to hold onto the complexity and locatedness of maternal subjectivity for mothers of children with disabilities.

## Introduction

Our approach in this study is, firstly, to develop a rationale for a psychosocial conceptualisation of disability by providing a brief outline and critique of two distinct areas of research or theorising relevant to childhood disability – the social model of disability and the attachment literature. We argue that these totally separate bodies of work point to the social–psychological dualism in the literature which maintains the false and unhelpful division between inner and outer worlds critiqued in an emerging field of research called psychosocial studies. This argument is not new within the field of disability where Shakespeare (2006a) has argued against dichotomous thinking and where Watermeyer and Swartz (2008) and Goodley (2011) have argued for the value of drawing together critical disability studies with social psychoanalytic thinking. However, drawing together the social model with contemporary attachment theory (as arguably a variant of psychoanalytic thought) is a novel version of this, perhaps a more risky one, but one that we argue is nevertheless important and productive.

In the second part of the study, we briefly outline a conceptualisation of subjectivity as psychosocial before thickening this description in the context of childhood disability through its application to a reading of interview segments with a mother of a disabled child. We try to demonstrate how such a conceptualisation attempts to hold onto the gains of both more traditional psychological research and more critical social research without reducing each to one part of a causal relationship. It is hoped that in this regard the study contributes towards ongoing work to develop sufficiently complex and yet pragmatic analytic methods for qualitative research in childhood disability studies. In particular, we argue that the strength of the analytic approach that we outline lies in its aims to both understand and disrupt subjectivity.

## The social versus the psychological

There has been much historical and contemporary theorising about the terms used to describe people with disabilities, emphasising the ways in which language is used for social and economic purposes. Linton (2006) describes the medical origins of the term disability and documents the reassignment of meaning to this term by disability rights activists, policy makers and healthcare professionals in order to reinterpret disability as a political category. This research draws on the International Classification of Functioning, Disability and Health (WHO 2001) to define disability in children and therefore moves away from a sole emphasis on individual limitations to include a socio-political definition that emphasises ‘society’s unwillingness or inability to accommodate the needs of all citizens, irrespective of bodily difference or impairment’ (Andrews, Fourie & Watson 2006:245). This social model of disability emphasises the assessment of the individual’s functioning within their physical, cultural and policy environments (Mont 2007), viewing disability as based in unequal social relationships (Connors & Stalker 2007) because of barriers to ‘doing’ and ‘being’ placed on people with disabilities by non-disabled people.

Despite the strides made in conceptualising disability as ‘an experience that arises out of the interaction between a person with a health condition and the context in which they live’ (Schneider 2006:8), too little has changed in relation to the discriminating environment that disabled people interact with. An interview study with 26 disabled children (with a variety of impairments) on their experiences of disability found that the children experienced disability in terms of impairment, difference, other people’s behaviour towards them and material barriers (Connors & Stalker 2007). Moreover, Maulik and Darmstadt (2007) established in their review of available research that research conducted in low- and middle-income countries continue to use screening measures that do not take into account functional limitations or degree of service utilisation or impairment of role performances, rather the disability is diagnosed medically which does not take into account the social model of disability. Furthermore, in a study of parents’ experiences, Kearney and Griffin (2001:587) emphasise the extent to which parents described the negative contribution of others to their experience of sorrow including professionals’ expressions of hopelessness highlighting that ‘developmental disability hold a multiplicity of negative meanings, resulting in society attitudes and practices that are dehumanizing’. It is clear from these selected research findings that the social model of disability still has a significant role to play in advocating for the rights of disabled people and in exposing the ableism so prevalent in society.

Nevertheless, for all the strengths of the social model, many of which still need to be realised in the South African context, the subjectivity of the disabled person tends to fall into the cracks between their impairment and their context. With the emphasis of the social model approach on discrimination and systematic exclusion, Watermeyer (2009) draws attention to a danger within this model of conceptualising prejudice as a lack of knowledge, overlooking the emotional pay offs of discrimination. Furthermore, such an emphasis has resulted in the subjectivities of disabled people and of caregivers of disabled children (the focus of this study) remaining relatively empty, ultimately contributing to the stereotypes that the social model of disability works so hard to challenge.

In sharp contrast to this exclusively social emphasis, within developmental psychology and mental health more generally there has over the past decade been an explosion of attachment-based research and interventions. Research has found that sensitive and responsive attunement by caregivers to infants’ and toddlers’ behaviours is associated with improved child development in non-disabled children (Shonkoff & Phillips 2000 cited in Dunst 2007) and in disabled children (Trivette 2003; Trivette 2004; Trivette & O’Herin 2006 all cited in Dunst 2007). This important finding has seen an increasing recognition of the importance of early interventions (0–5 years) for overall development and mental health, where early intervention is defined as the experiential opportunities provided infants and toddlers with disabilities by the children’s parents and other primary caregivers that are intended to promote the children’s behaviour competencies, shaping and influencing their prosocial interactions with people and objects (Dunst 2007). Significantly, this definition explicitly involves parents and other primary caregivers as children’s primary sources of learning and therefore interventions are directed at increasing parents’ capacity to provide their children with learning experiences as well as children’s capacities to engage with self-directed and self-initiated interactions with others and with their environment. The individual and relational emphasis of this approach is explicit and sharply distinct from the social model of disability.

The sensitive and responsive attunement by caregivers that is being promoted in these early intervention programmes is based on attachment research, which is now well established, that identifies three patterns of attachment, namely secure, insecure and insecure ambivalent (Ainsworth *et al*. 1978), which are associated with different developmental outcomes. According to Howe (2006), children whose primary attachment relationship is classified as secure experience their caregiver as sensitively attuned in their caregiving, leading to greater social competence and more satisfactory relationships. By contrast, children whose primary attachment relationship is classified as insecure have been found to minimise affect in their relationships with others and develop hypervigilance, spending less time exploring their environments and more time monitoring their safety and security. Making the relationship between child and caregiver central in childhood disability interventions is considered important because attachment research points to a number of factors that increase the risk of less secure attachments forming between parents and children with disabilities including the more demanding and potentially stressful nature of caregiving (Howe 2006). Mobarak *et al*. (2000) found 41.8% of mothers living in rural areas in Bangladesh and caring for severely physically disabled children at risk for psychiatric morbidity. In particular, child behaviour problems (sleep problems, bed wetting, soiling and hyperactivity) were significant predictors of maternal stress. Howe (2006:750) posits that ‘children in need of particularly sensitive caregiving might challenge the parents’ ability to provide such care’ partly because of facial, postural and vocal behaviour typical in infants with developmental delays that make it more difficult for caregivers to read their signals and needs and respond appropriately.

From this attachment perspective, an explicit aim of early interventions for both non-disabled and disabled infants and their caregivers is therefore to support the parent–child relationship, and this is done by focusing on something called ‘mentalising’. Mentalising is ‘attending to states of mind in oneself and others’ (Allen 2006:3) and is associated with secure attachments that are in turn associated with social, emotional and cognitive benefits. More specifically, the aim of early interventions is to facilitate more secure attachments through increasing caregivers’ reflective functioning, which is defined as ‘an individual’s capacity to mentalise, that is, to envision mental states in the self o the other’ (Slade 2007:641). Certainly, such an approach recognises that it is not disability per se that predicts increased risk of maltreatment or insecure attachment. Rather it is the interaction between firstly the state of mind of the caregiver and secondly the infant (both non-disabled and disabled) who brings something to the relationship (grumpy temperaments, a particular type of impairment) in terms of its capacity to activate heightened arousal in the caregiver that works against reflective functioning (Howe 2006). However, the attachment approach’s emphasis on individual capacities and relational attunement narrows the focus of attention to *inside* the attachment context. Ignoring relationships and environments *outside* of the attachment context elides the ways in which they are likely to create or shut down conditions of open attention and social safeness that facilitate mentalising (Liotti & Gilbert 2011), conditions that the social model of disability brings into sharp focus. While the attachment literature certainly takes as its focus the complex social interactions in which children and mothers must engage, a critique of the medical model of disability (McKenzie & Muller 2006:313), the focus on increasing mothers’ capacities for reflective functioning nevertheless contributes to the message that ‘to have a disability (or to have a disabled child) is to be somehow incomplete and perpetually in need of help’.

## A psychosocial theoretical framework

Thus far we have outlined literature that either emphasises the social at the risk of emptying out the subjectivity of disabled people and their caregivers or emphasises relational and individual capacities of caregiver–child relationships at the risk of ignoring the broader social context, which provide the conditions for this relationship. Such a division between inner and outer worlds fails to account for the complexity of lived experience, and such accounting is necessary to do justice to the qualitative data that we collect in our research as well as to the human experiences we encounter in our therapeutic work. An alternative to the social-psychological dualism described thus far lies in an emerging field of research called psychosocial studies, which argues that deconstructing social discourses to understand and challenge ableism only takes us part of the way. While applauding the social model for excavating the social, economic and political processes linked to discrimination against disabled children and their caregivers, a psychosocial approach is also concerned with the extent to which purely discursive explanations are limited by their inability to conceptualise disableism ‘in its least “signifiable” aspects’ (Hook 2006:209).

A psychosocial perspective, while not at odds with the critical realist perspective espoused by Shakespeare (2006b), which conceptualises disability as the interaction between individual and structural factors, emphasises that this interaction is such that it is neither possible nor helpful to continue to think about individual factors as being in the person and structural factors as being outside the person. Rather, inside and outside are two sides of the same coin; what one considers to be ‘outside’ can also conceivably be thought about as ‘inside’. The moebius strip, a surface with only one side and only one boundary, is frequently evoked as a metaphor for the way in which the psychological and the social are ‘underside and topside’ of the same thing (Frosh & Baraitser 2008:349).

Psychoanalysis, as a body of theory has been consistently drawn upon in psychosocial work with its emphasis on language and the construction of meaning through language as a point of continuity with discursive work. What psychoanalysis brings to discursive work is an emphasis on the affective realm and a rich vocabulary for capturing intersubjective encounters without limiting the analytic gaze to only what is said. Rather, what is absent and yet structuring of the present is equally important in understanding subjectivity. Psychoanalysis, with its emphasis on the unconscious as that which always hides and yet finds expression in some altered form (Frosh 2002), has been an important way of addressing critiques of discursive work as devoid of the psychological so that one is left with ‘empty’ subjects that parrot social meanings. Most importantly, the concept of the social unconscious (Hopper 2003) is also what psychoanalysis brings to the picture, a complex concept described by Hopper (2001, cited in Weinberg 2007:311) as:

the existence and constraints of social, cultural and communicational arrangements of which people are unaware. Unaware, in so far as these arrangements are not perceived (not ‘known’), and if perceived not acknowledged (‘denied’), and if acknowledged, not taken as problematic (‘given’), and if taken as problematic, not considered with an optimal degree of detachment and objectivity.

This position is not dissimilar to Goodley’s (2011:716) reference to the ‘cultural and political foundations of the psyche’. In addition, one could argue that the social unconscious operates through the invisible labour referred to by Kittay (2009:2) ‘done by people with disabilities and their families to allow those without disabilities to understand and interact with people with disabilities’. Furthermore, the social unconscious can be seen to operate in the multiple ways that dependency work remains invisible while Kittay (1998:127) argues ‘those who are unable to take advantage of new opportunities are blamed for their own distress’. It is these insidious, less visible and least signifiable aspects of our social worlds that require and resist our attention.

For the purposes of this study, we draw on contemporary attachment theory (Fonagy *et al*. 2002) as our psychoanalytic paradigm alongside discourse theory (Potter & Wetherell 1987) to conceptualise the psychosocial subject, with both paradigms sharing a concern with language or communication as the medium through which people compose themselves. It is essential to acknowledge at the outset that in the body of work that represents contemporary attachment theory, there are conservative pulls and therefore it is important to state that in this study we endeavour to reject the normative use of attachment theory and strive towards employing its more critical edge, which is frequently at its points of dialogue with psychoanalytic theory. (A discussion of the similarities and differences between contemporary attachment theory and psychoanalysis is beyond the scope of this paper, but see Fonagy 2000).

We are interested in the extent to which a contemporary attachment theoretical framework, in particular, is conducive to a rethinking of the subject–other relation, one that rejects an a priori division between inner and outer worlds outlined above and possibly allows for some ‘rehabilitation’ of the social model of disability. Central to this endeavour is the concept of mentalisation, which is defined as a process of imaginative mental activity that involves perceiving and interpreting the intentional mental states of ourselves and of others (Fonagy *et al*. 2002). This capacity to mentalise includes the ability to recognise the opacity of others’ minds, which is central to resisting stereotyping so prevalent in prejudice (Twemlow & Fonagy 2006; Twemlow & Sacco 2007). Rather than being a cognitive rational concept, mentalisation is conceptualised as an intersubjective concept that evolves in the affective tie to others (Diamond & Marrone 2003). While this affective tie has been largely conceptualised at the level of the caregiver–child relationship, increasingly researchers have begun to recognise that a sense of security which facilitates mentalisation has sources outside of the attachment system (Liotti & Gilbert 2011) such that the socio-historical context/‘then and there’ (Parker 2014) and the interpersonal context/‘here and now’ has been shown to facilitate and/or restrict the capacity to mentalise or the ability to hold the other in mind (Fonagy & Higgitt 2007). Acknowledging the multiple contexts which might provide social safeness is very important because it reconceptualises mentalisation capacities as context dependent, as situated *between* persons and *between* persons and their contexts, rather than as a stable mental trait inside individuals’ heads (Liotti & Gilbert 2011).

There is a further quality to mentalising that enables a critical use of contemporary attachment theory: the extent to which the opacity of the mind is upheld. An essential quality to holding someone’s mind in mind is recognising that one can never really know the other. In this regard, we might think of the social model as playing a significant role in upholding the opacity of the mind, as Kittay (2011) argues that the role of the social model of disability is to force parents to see that they cannot see the world from their disabled children’s perspective. In this sense, mentalisation might be conceptualised as striving both to understand and disrupt. In mentalising, we think about our own and others’ states of mind in a way that increases meaningful connection *and* we also always allow the possibility for disjointedness, for being caught unawares by the other. It is the movement between understanding and disruption that allows the mind to retain its opacity and which gives mentalisation a quality that is not dissimilar to the dynamic unconscious.

From this perspective, perhaps we are not entirely in agreement with Shakespeare’s (2006b) rejection of the social model, while we recognise its constraints, we want to hold onto the interruptions it provides alongside attempts to interpret. In his argument against the social model, Shakespeare (2006b) refers to its disregard for different levels of impairment and refers to Vehmas’ (2006 cited in Shakespeare 2006b) writing about Steve, a man with severe intellectual impairment, a man who because of the degree of his impairment, would not benefit from initiatives like independent living, barrier removal or civil rights that the social model offers. While we do not disagree with Shakespeare and Vehmas regarding the level of impairment being an important consideration and with the claim that some impairments are more limiting than others, we do have questions about whether Steve is unlikely to benefit from the social initiatives described, even if indirectly. Kittay’s writing is informative here as she considers those who Steve is dependent upon as central to how we think about Steve. Certainly, from Kittay’s perspective, the kinds of initiatives described by Vehmas embodied by the social model are likely to have an impact on Steve’s dependency workers whether they be a paid caregiver or parent (Kittay 2011). Specifically, they are likely to provide these dependency workers with a sense that their dependency work is important, valued and part of a broader movement to increase the visibility of disability. There is a disruption that is provided for dependency workers that renders *them* more visible by association, even for a short while.

To summarise, we have above been describing a psychosocial theoretical perspective from which to view subjectivity in disability studies. Such a perspective attempts to blur the boundaries between the social and the psychic and argues that psychoanalysis can be usefully employed in this regard to see the ‘we’ and the ‘I’ as inseparably connected, as not joined together but as never individually conceived. We have extended this discussion with the concept of mentalisation as providing us with a means to both understand and disrupt ourselves and others in context. It is these opposing movements of understanding and disruption, or interpretation and interruption that the analysis below aims for in putting the concept of mentalisation to work.

## A psychosocial analysis: Reading the encounter

The methodological approach employed is binocular in its emphasis on both top-down interpretations, employing discourses and mentalisation as central theoretical concepts, alongside a bottom-up approach that grounds interpretations in the text (its content and structure) and in the research encounter (non-verbal aspects of the relational field, captured directly after the interview but also present in the analysis). Such an approach has been conceptualised as a concentric reflexive approach (Saville Young & Frosh 2010) consisting of multiple readings of the text and research encounter, with each reading stepping outside of the previous one in a concentric outward movement. Such an approach recognises that one is always constrained by one’s perspective, and therefore argues that interpretations are necessarily tentative; we hope to understand and then to disrupt our understandings.

For the purposes of this study, we would like to focus on a particular fairly lengthy extract, which is taken from an interview that Jessie conducted as part of a broader research project on caregiving of disabled children. Jessie is a white woman and at the time of conducting the research was a psychology student in her early twenties, and the participant, Irene, is a white, non-disabled woman employed as a secretary, who is also a mother to a 13-month-old non-disabled daughter and a 6-year-old son with physical and developmental disabilities. The following extract has been chosen for its emotional content in particular pointing to active meaning making on behalf of both Irene and Jessie:

J: ‘What about other sorts of stress, you’ve mentioned that you’re worried about losing your job.’

I: ‘Ya um I take him to therapy once a week and my bosses are getting a bit tired (J: mm) of me taking time off ‘cause I’m a receptionist and I can’t really take time off but I’ve got an understanding boss, they’ve both got kids (mm) so it’s just one day they might have enough, say look you can’t do this anymore, thank you goodbye (mm) basically what I’m stressing about at the moment ‘cause we’ve got to take him to PE in October for an eye op and well eye test (mm) and see the paediatrician (…) and that’s also a bit stressing me out at the moment, we don’t know what the eye doctor will say about his eyes that the main because he does have a bit of a squint (okay) (…)’

J: ‘Sorry I was going to ask you something and it totally slipped my mind now (embarrassed, flustered). So how do you deal with that stress?’

I: ‘(…) I shout, get very frustrated and I take it out on the people closest to me (…) which I shouldn’t, I should rather step away from the situation, go for a walk (mm) but instead of that I take it out on the people nearest and dearest to me (…)’

J: ‘I’m sure that makes everything a lot more complicated.’

I: ‘Yes, a lot more difficult so I shouldn’t, should rather try and be calm (mhmm) instead of shouting and (…)’

J: ‘But that’s hard sometimes.’

I: ‘It is hard sometimes but I’m getting there (…)’

J: ‘(…) Sorry I just think we’ve gone through everything quite quickly (…) is there anything that you’d like to add?’

I: ‘No, nothing at the moment, I’m just grateful for people that reach out to us (mm) that are there so that we’re not alone because that’s how I feel (…) alone, I might have that support group but I still feel alone, still feel that (…) why me, why do I have to get through this (very emotional, she starts crying) (oh no, don’t worry) (…) We love James (ya) but we had so many dreams and aspirations for him and now we’ve got to have different dreams, different aspirations for him, different different life than Catherine for instance (mm) I feel guilty that she is perfect, nothing wrong with her and look at James, why what does things like this happen to us (…) (mm) we’ll never know (…)’

J: ‘Are you religious at all?’

I: ‘Yes very, I’m very involved in my church and (..) I’ve asked questions but I’m still waiting for answers (mm) some answers have been, some questions have been answered but there’s just a few questions that we’re still waiting to hear the answer for.’

J: ‘Like what kinds of questions?’

I: ‘Um (…) research, would there be any, could they do any research to find out what caused what causes autism (ya) why is there such a thing as autism, why are the numbers so high it’s and you read books and eventually you get to a certain page in the book and you just close the book and you put it away because you don’t want to read anymore, it gets you more anxious, more upset (mm) more cross that you could’ve maybe prevented it, what went wrong (…).’

The first level of analysis concerns the discursive positions that are taken up and resisted in the text. We are interested in how discourses define and limit what can and cannot be said, not only because these are *disallowed* by dominant shared meanings of motherhood and disability but also because they are *disavowed* at a societal level. This reading is in line with a deconstructionist, disruptive stance not incompatible with the social model of disability (for further reading on this analytic stance, see Saville Young 2014).

In this extract, we see Irene draw on a medical discourse, prominent throughout her interview, firstly in her listing of tests and specialists and impairments associated with James (l. 7–10), and secondly in her appeal to research to answer questions and provide expertise (l. 37–39). This medical discourse constructs James as an ill patient with various parts of himself that are dysfunctional (his eyes, his mind) requiring treatment by experts. This medical discourse is fed into by a discourse of perfection which is drawn on to construct her daughter as a non-disabled child in contrast to James (l. 29–30). The effect is a smoothing over of the struggles and difficulties of non-disabled children such that what is difficult and hard belongs entirely and fully with disabled children like James contributing to a stereotyped, one-dimensional view of disabled subjectivity. One could argue from this perspective that Irene’s feelings of guilt (l. 29) are constructions produced by the effect of these two discourses, which suggest that something *has* gone very particularly wrong with James and therefore disability is blameworthy. Locating these effects in an emotional state can be seen to contribute to an individualisation of motherhood (as something that individuals do well or do not do well) and by extension of disability. The moral judgement synonymous with this individualisation of maternity is further read out in Irene’s description of herself as getting ‘very frustrated … which I shouldn’t’ (l. 13). While the text has moments of Irene being somewhat kinder to herself [‘but I’m getting there’ (l. 20)] and to James [‘we’ve got to have different dreams, different aspirations’ (l. 28–29)], these are in effect simply positive versions of disability and caregiving within the same framework. Therefore, they work to validate the individualised moral framework rather than resist it.

A discourse of loss is prominent in the text constructing Irene as giving up the position of being a mother with ‘dreams and aspirations’ (l. 28) for her son, taking up a position of difference (l. 28–29). Her description of having ‘had so much dreams and aspirations for him’ (l. 27–28) construct herself as being duped into a sense of normality, only to have it taken from her and replaced with difference. The narrated experience here is one of loss that is built on an ideology of the knowledgeable or knowing mother particularly prevalent in contemporary society where parenting books and classes abound. Against this particularly oppressive mothering ideology (see Ryan & Runswick-Cole 2008) that maintains the notion that perfect babies and children can be made through knowledgeable parenting, Irene, as the mother of a disabled child is constructed as unknowing, left asking ‘why, what, does things like this happen to us … we’ll never know’ (l. 30–31).

The above discursive reading points to the work that a social model of disability is called on to do – to challenge the location of disability in the individual, as well as the construction of disability as quite separate from the non-disabled experience. However, it is important not only to pay attention to how the role of disabling society is disallowed through such talk but also to how it is disavowed – it is not just absent, rather its presence is actively resisted. From this perspective, we see how Irene has an ‘understanding boss’ (l. 5) and that the difficulty she finds herself in (located in personal stress) is not because of these bosses who are parents themselves but in the perfectly reasonable position that receptionists cannot take time off work (l. 4). The insidious way in which ‘dependency work’ (Kittay 1998) is rendered invisible elides its resistance to being known.

We now move to the second tier of analysis in which we move to understand Irene’s emotional investments in the construction of ‘not knowing’ specifically. In doing so, we draw attention away from socially shared discourses to a thinking through the emotional pay offs or the conscious and unconscious ‘reasons’ for their employment in order to not lose sight of Irene, a particular mother of a particular disabled child. In the extract above, Irene describes the ambivalence of wanting to know the cause of autism, of ‘waiting’ (l. 34) to ‘hear the answer’ (l. 35) and also not wanting to know the cause ‘you just close the book and you put it away’ (l. 40). This could be read symbolically as a defensive denial of her own sense of guilt in relation to James. We might understand that Irene, while frustrated with not knowing, is also at the same time invested in not knowing because she does not want to really know or face her own sense of being responsible (earlier in the interview she makes reference to only going to the doctor when she was 6 months pregnant). Employing the concept of mentalizstion, we might argue that Irene’s stance above represents an anti-mentalising stance. She does not want to know her own mind, which already is certain of her own culpability. The problem with this reading is that it psychologises Irene, the analysis itself contributes to the individualising medical and moral discourse that the discursive reading was so critical of. Rather than employing a mentalising or non-mentalising capacity internally located in Irene, we want to argue that specific affective processes enable Irene’s investment in this position of not knowing, in this anti-mentalising stance and that these affective processes do not belong to Irene but are rooted in this particular intersubjective exchange (‘here and now’) and the particular socio-history context (‘then and there’) in which she finds herself.

In order to do this, we turn to the symbolic, emotional and enactive representations specific to the particular interpersonal encounter of the research interview; we locate the text in its *research* context in a reflexive disruptive move. To capture these representations, the interviewer, Jessie, wrote a narrative that detailed her feelings and thoughts about the interview with Irene, those that arose during the interview as well as those that have arisen subsequently. This narrative formed part of the data and informed the analysis. However, it is important to acknowledge that, while these representations are certainly grounded in the text, they are also part of the reconstruction by the interviewer and therefore reflect her subjective experience of the encounter, thus embracing the psychoanalytic notion of understanding as depending on ‘the subjective exploration of one person by another’ (Frosh & Saville Young 2008:115). A significant aspect of these reflexive notes was Jessie’s experience of losing her own mind. Jessie describes in her notes struggling to hold onto her thoughts during the interview. These feelings are reflected in the data as she forgets and questions slip from her mind (l. 11 and l. 22). Part of this struggle was rooted in the heavy emotional quality of the interview, which was punctuated by lengthy silences from Irene which Jessie experienced as highly defensive – as if Irene felt the need to protect herself from the interview questions, perhaps again not really wanting to know what was in her own mind. But part of this struggle may also be rooted in Jessie’s own investment in not wanting to really know Irene as a holistic and multi-dimensional mother, but rather already feeling pity and anguish for her in a stereotypical view of a mother of a disabled child that fails to acknowledge the positive experiences of being a parent of a child with a disability (see Kearney & Griffin 2001).

This interpretation of the ‘here and now’ is underscored by a further interpersonal process, which sees Jessie introduce religion into the conversation (l. 32). On the one hand, this could be read as Jessie introducing a stereotypical view of mothers of disabled children as saints (James 2012). This religious model of disability echoes the sentiment that God chooses which mother will have which child (Bombeck cited in James 2012), suggesting that it is the mother’s individual characteristics that determine her ability to care for a disabled child. On the other hand, we might read into this introduction of religion as belying Jessie’s difficulty in managing her own anxiety, faced with Irene’s narrative of struggling to mother her disabled son. It is almost as if Jessie is calling on religion to help her in this particular situation in ways that disabled people, and in this case mothers of disabled children, are very used to having to manage. In ways that have been documented by Watermeyer and Swartz (2008:601) in relation to the experience of disabled people and referred to as a ‘disguising of experience’ and a ‘dampening down of authentic responses’, similarly Irene can be seen throughout the interview to try to be appeasing and untroublesome [‘I’m getting there’,(l. 20) and ‘I’m grateful to people that reach out to us’ (l. 24)].

Drawing attention to these processes occurring in and around Jessie and Irene is not meant to berate Jessie for her prejudice but rather to point to the common ways in which the emotional work we engage in to understand and empathise, given the anxiety-provoking nature of this work, can in effect restrain our empathic ability. From this perspective, we are arguing that Jessie is holding Irene in mind in a particular way: as someone who is deserving of pity (needs godly help) and/or as a ‘saint’; she is not curious about who she is but seems to already have pinned her down from outside. Importantly, this can be understood as not necessarily only belonging to Jessie but rather as reflecting an unconscious historical group identity of ableism (‘there and then’), which represents a fantasy of mastery, completeness and perfection that is so dependent on the projection of loss onto others (Watermeyer 2009).

The principle of mentalising begets mentalising is here worked out in the negative; this reading draws attention to Jessie as a bystander (outside of Irene and James’ relationship in which we might interpret her as adopting an anti-mentalising stance) but nevertheless actively fuelling the way in which Irene is not able to hold James in mind through her inability to attend to her own mind and that of Irene’s with a quality of openness. This interpretation resists placing the emotional investment in the discourse of ‘not knowing’ within Irene’s psyche; rather, the analysis seeks to understand investments as always already belonging in, around, between and outside Irene. This draws attention to the dynamic and shifting nature of Irene’s subjectivity, to her capacity to mentalise potentially waxing and waning in relation to the container, which Bion (1985) conceptualised as needing to take up and absorb the consequences of raw emotional experience. This view of her maternal subjectivity is significantly not fixed, nor is it entirely dependent on her inner capacities.

The final tier of analysis is to think about what the reading thus far does – what does it produce – and also by what the analysis is constrained. This final tier tries again to capture the analytic emphasis on understanding and then disrupting. With respect to how it moves our understanding along, the above reading challenges notions described by James (2012) that construct mothers of disabled children as saints, rather Irene’s narrative points to someone who struggles with mothering her disabled child. While not always comfortable, drawing attention to these moments is important in order to present mothers of disabled children as subjects with complex and ambiguous feelings that dynamically shift throughout the interview from acceptance to non-acceptance, from loving to rejecting. In this way, Irene begins to resemble more critical depictions of all mothers as both selfish and selfless (James 2012), resulting in a multi-dimensional subjectivity that is holistic and constantly in flux. Therefore, the above reading contributes to work on maternal subjectivity within disability studies challenging idealistic accounts of mothering, encouraging diversity in relation to the maternal position with an emphasis on the dynamics of mothering as opposed to a constant status and with an emphasis on local manifestations of mothering as opposed to universalising tropes.

However, perhaps the analysis is constrained by its use of mentalisation in a conservative pull that we need to be mindful of, a pull that might suggest that Jessie could have conducted the interview ‘better’. Specifically, we want to argue that it is important to recognise the inevitability of Jessie’s position in the encounter above. This inevitability of the researcher’s position seems to be unacknowledged in many of the mentalisation interventions aimed at supporting mothers of infants, both non-disabled and disabled, to mentalise (e.g. Sadler, Slade & Mayes 2006). There is a tendency in this interventionist work to take the clinician out of the encounter, to disregard the atmosphere of observation that she or he is contributing to which in turn facilitates or shuts down potentialities for mentalising. Research suggests that these interventions are highly successful (see Sadler *et al*. 2013), and we do not want to disregard this important work. Nevertheless, what is often missing is the social perspective, represented by the clinician who despite his/her best intentions will not be able to eradicate their prejudice, being on the side of the intervention, exemplified in their reaching for a secure attachment between caregiver and child. In doing so, there is a danger of not being able to recognise ‘the unbearable knowledge’ (Frosh 2011:235) that we are always, inevitably and to some extent, contributors to insecurity and that the best we can do is attempt to step outside of ourselves to disrupt what we think we know. This final tier of analysis asks whether attachment theory, with its recent proliferation of measurements of reflective functioning and interventions, is able to tolerate our hateful feelings towards our (disabled and non-disabled) children and their mothers to promote an environment of inclusion.

## Reprise

The reading above draws attention to discourses that position Irene and her children in particular ways while also pointing to investments in these discourses such that these discourses are not purely social but play affective functions. Significantly, our reading places these affective functions between and around Irene and the interviewer; they move through these spaces in ways that are both revealing and resistive to being known. We use multiple lenses to read the text, seeking to understand what is going on from within each lens (discursive/social, interpersonal, intrapsychic), while also seeking to disrupt this understanding as we take up the position of a different lens. The concept of mentalisation is employed throughout in different ways – always seeking to both understand and hold onto the impossibility of doing so.

While this study has chiefly been concerned with conceptualising a psychosocial conceptualisation of subjectivity that employs discursive psychology alongside contemporary attachment theory and putting this to work methodologically, this study has also given voice to Irene in a particular context. There is a concern that retelling difficult stories such as that of Irene plays into a voyeuristic interest in tragic stories of disability (Goodley & Runswick-Cole 2013), which must be acknowledged while holding onto the importance of giving a voice to narratives of mothers of disabled children whose dependency work is so often invisible (Kittay 1998) in order to unsettle taken-for-granted notions of mothering and disability. In the same way as Shakespeare (2006b) in his book, ‘Disability Rights and Wrongs’, critiques the social model of disability for failing to adequately account for the pain and limitation of impairments, so we must be careful of idealising caregivers and dependency work, being sure to look at things that are hard to look at. Irene provides us with this opportunity. Struggling with the impairments of one’s children is an important aspect of caregivers’ lives. In order to integrate them, they must be acknowledged but they must also not be located solely in these mothers – we hope that our analysis goes someway to highlight the ways in which Irene’s story both does and does not belong to her. Drawing attention to the inextricability of the personal and the social such that what is ‘in the mind’ can only be thought under particular circumstances reminds us all that in our personal meditations, interpersonal interactions and social endeavours to remain committed to challenging disabling ways of robbing mothers of disabled children of the complexity and locatedness of their maternal subjectivity.
